# Modeling brain metastases in cost effectiveness analysis of atezolizumab for extensive stage small cell lung cancer

**DOI:** 10.1038/s41598-025-22966-4

**Published:** 2025-11-10

**Authors:** Hsiao-Ling Chen, Chen-Han Chueh, Wei-Ming Huang, Shu-Hua Chan, Hsiao-Hsiang Cheng, Shao-Chin Chiang, Chiao-En Wu, Yu-Wen Wen, Yi-Wen Tsai

**Affiliations:** 1 Institute of Health and Welfare Policy, National Yang Ming Chiao Tung University, No. 155, Sec.2, Linong Street, Taipei City, Taiwan; 2 Herbert Wertheim School of Public Health and Human Longevity Science, University of California San Diego, La Jolla, San Diego, CA USA; 3 Medical AI Development Center, Taipei Veterans General Hospital, Taipei, Taiwan; 4 Department of Pharmacy, National Yang Ming Chiao Tung University, Taipei, Taiwan; 5 Department of Oncology and Hematology, Koo Foundation Sun Yat-Sen Cancer Center, Taipei, Taiwan; 6 Department of Pharmacy, Koo Foundation Sun Yat-Sen Cancer Center, Taipei, Taiwan; 7 Division of Hematology-Oncology, Department of Internal Medicine, Chang Gung Memorial Hospital at Linkou, Chang Gung University College of Medicine, Taoyuan, Taiwan; 8 Division of Hematology-Oncology, Department of Internal Medicine, New Taipei Municipal TuCheng Hospital, New Taipei, Taiwan; 9 Department of Biomedical Sciences, Chang Gung University, No.259, Wenhua 1st Rd., Guishan Dist, Taoyuan City, Taiwan

**Keywords:** Atezolizumab, Brain metastases, Cost-effectiveness analysis, Extensive-stage small cell lung cancer, Cancer, Oncology

## Abstract

Brain metastases (BM) significantly impact prognosis and healthcare utilization but remain underrepresented in economic evaluations. The IMpower133 trial demonstrated the efficacy of atezolizumab plus chemotherapy (carboplatin and etoposide), particularly among patients without baseline BM, leading to its approval for extensive-stage small-cell lung cancer (EX-SCLC) in several countries. As previous studies did not incorporate BM status, we addressed this gap by including BM in the cost-effectiveness analysis of atezolizumab for EX-SCLC. A four-state Markov model was constructed with the following health states: progression-free, progressive disease (PD) without brain metastases, PD with brain metastases, and death, over a 15-year time horizon. Time-varying efficacy and utility were derived from IMpower133; direct medical costs were estimated using population-based databases. Both deterministic and probabilistic sensitivity analyses were conducted to assess uncertainty in input parameters. Adding atezolizumab to chemotherapy increased QALYs by 0.721 at an incremental cost of NT$2,111,359, yielding an incremental net monetary benefit of NT$68,264, and a 53.22% probability of cost-effectiveness. Key sources of uncertainty include efficacy, cost of atezolizumab, time horizon, and utility. In conclusion, adding atezolizumab to chemotherapy is cost-effective for patients with EX-SCLC without baseline BM. Incorporating BM status into CEA could support more nuanced policy recommendations.

**Supplementary Information:**

The online version contains supplementary material available at 10.1038/s41598-025-22966-4.

## Introduction

 Small cell lung cancer (SCLC) is often asymptomatic in its early stages but progresses rapidly, with > 70% of cases diagnosed at the extensive stage (EX-SCLC)^1^. Although standard chemotherapy with etoposide and platinum helps achieve a 60–80% overall response rate^2^, most EX-SCLC patients experience relapse within 6 months, and the 5-year survival rate remains around 2%^3^. This poor prognosis highlights the urgent need for novel therapeutic approaches.

Atezolizumab, an immune checkpoint inhibitor, demonstrated promising results for EX-SCLC in the phase III IMpower133 trial. Adding atezolizumab to chemotherapy (carboplatin and etoposide) improved overall survival (hazard ratio [HR] = 0.76, 95% confidence interval [CI] = 0.60–0.95) and progression-free survival (HR = 0.77, 95% CI = 0.62–0.96) in treatment-naïve patients^4,5^. However, its high cost presents a major challenge, with incremental cost-effectiveness ratios (ICERs) of $528,810 per quality-adjusted life-year (QALY) in the U.S. and $489,013 per QALY in China, both exceeding their pre-defined willingness-to-pay (WTP) thresholds. Low cost-effectiveness probabilities (0–6%) underscore inefficiencies in insurance reimbursement, thereby limiting patient access to this innovative treatment^6–10^.

Previous cost-effectiveness analyses (CEAs) considered the EX-SCLC population as homogeneous, overlooking patient heterogeneity in response to the addition of atezolizumab to chemotherapy^6–10^. Notably, brain metastasis (BM) is the most critical among the key sources of heterogeneity owing to its critical impact on treatment outcomes^11^. Data from the IMpower133 trial^4,5^ revealed that patients without baseline BM benefited from the addition of atezolizumab, with improved overall survival (HR = 0.74, 95% CI = 0.58–0.94). Based on observed clinical benefits, Taiwan’s National Health Insurance (NHI), a single-payer universal health coverage system, began reimbursing the combination of atezolizumab and chemotherapy for patients with EX-SCLC without BM in 2023, even though a cost-effectiveness analysis (CEA) for this population had not been conducted. Nevertheless, performing a formal CEA remains essential, as evidence positions this regimen in the “more costly but more effective” quadrant of the cost-effectiveness plane. In contrast, the benefit for patients with baseline BM was less certain due to the limited sample size (HR = 0.96, 95% CI = 0.46–2.01), suggesting that economic evaluation for this population should be deferred until more robust and reliable evidence on clinical efficacy becomes available.

Beyond efficacy, BM contributes to heterogeneity in both quality of life and healthcare costs^12,13^, factors central to CEAs that have not been adequately incorporated into existing economic evaluation models. To address this gap, we developed a new four-state disease model for EX-SCLC that more accurately reflects real-world clinical scenarios by allowing for the natural progression and development of BM. Using this model, we evaluated the cost-effectiveness of atezolizumab plus chemotherapy as first-line therapy among patients with EX-SCLC without baseline BM, from the perspective of Taiwan’s National Health Insurance Administration (NHIA).

## Results

### Base-case analysis

As shown in Table 1, the 15-year simulation demonstrated that adding atezolizumab to chemotherapy resulted in a gain of 0.721 QALYs at an additional cost of NT$2,111,359. This resulted in an ICER of NT$2,928,376 per QALY, which was below the WTP threshold, and a positive INMB of NT$68,264. When LYs were considered as a measure of effectiveness, the ICER was NT$2,566,151 per LY, with an associated INMB of NT$375,928.

**Table 1:**
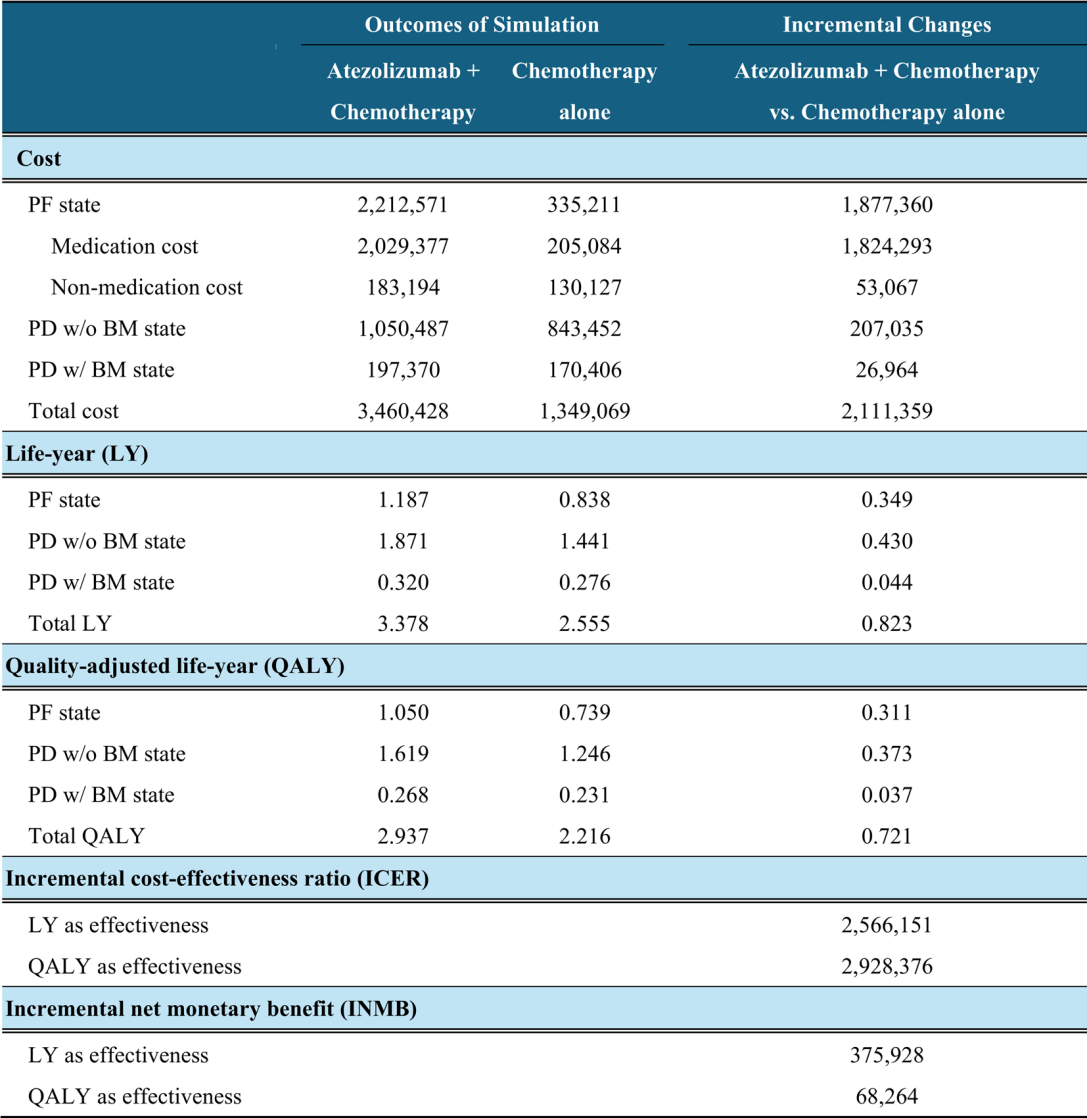
Results of the base-case analysis. PF: progression-free. PD w/o BM: progressed disease without brain metastases. PD w/ BM: progressed disease with brain metastases. Costs listed in New Taiwan Dollar (NTD).Medication cost: include cost from atezolizumab and chemotherapy. non-Medication cost: include cost form surgery, therapy for adverse events, radiotherapy and other health resource cost for SCLC. PD state cost includes subsequent therapy, surgery, radiotherapy and other health resource cost for SCLC.

PF: progression-free. PD w/o BM: progressed disease without brain metastases. PD w/ BM: progressed disease with brain metastases. Costs listed in New Taiwan Dollar (NTD).

Medication cost: include cost from atezolizumab and chemotherapy. non-Medication cost: include cost form surgery, therapy for adverse events, radiotherapy and other health resource cost for SCLC. PD state cost includes subsequent therapy, surgery, radiotherapy and other health resource cost for SCLC.

### Base-case sensitivity analysis

**Fig. 1:**
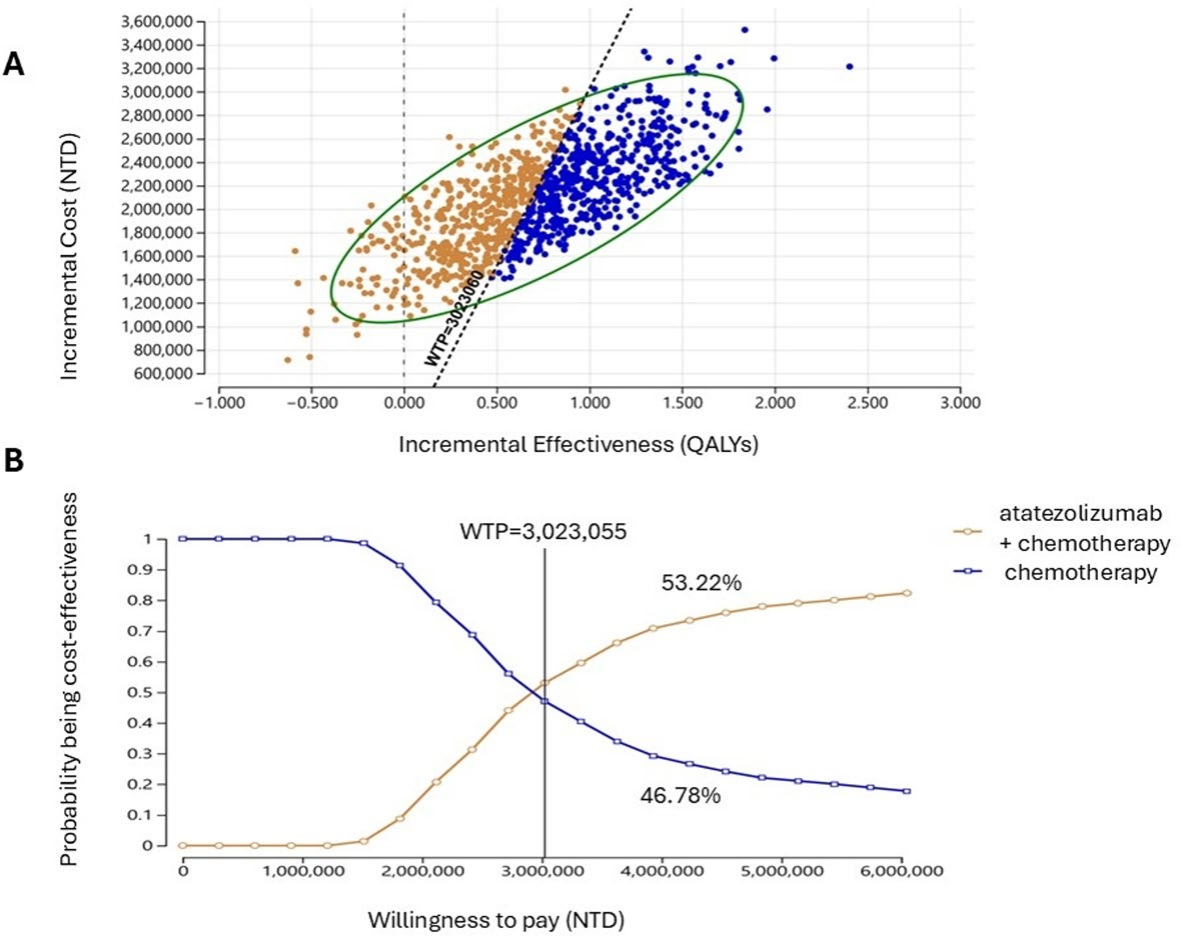
Probabilistic sensitivity analysis results. (**A**) Incremental cost-effectiveness plane and (**B**) cost-effectiveness acceptance curve for atezolizumab plus chemotherapy versus chemotherapy. The dashed line indicates the willingness-to-pay (WTP) threshold of NT$3,023,055 per quality-adjusted life year (QALY) gained. Costs listed in 2023 New Taiwan dollars (NTD).

Figure 1A illustrates the results of 5,000 simulation iterations on the cost-effectiveness plane. The PSA results showed that 95.00% of the iterations fell within the northeastern quadrant, indicating that combining atezolizumab with chemotherapy resulted in greater effectiveness at a higher cost than chemotherapy alone. The probability of the intervention being cost-effective was 53.22% (Fig. 1B). The tornado diagrams in Supplement 1 A and Supplement 1B present the DSA results for ICER and INMB, incorporating all parameters, including both clinical and cost variables, to comprehensively assess their impact on model outcomes. The most influential parameters affecting both ICER and INMB include variations in efficacy, price of atezolizumab, and utility of PD without BM. Among the efficacy parameters, the transition probabilities from PF to death and from PD without BM to death significantly affected CEA results in both treatment arms. Additionally, the time horizon has a substantial influence.

### Scenario analysis

The nine scenarios are listed in Table 2. Scenarios 1–3 focused on examining the impact of clinical efficacy on CEA results. Both Scenario 1 (second-best-fit model) and Scenario 2 (optimistic model) slightly increased the probability of cost-effectiveness, with smaller EVPIs than those of the base-case model. In Scenario 3, when pessimistic models were used, the intervention was not considered cost-effective. The probability of cost-effectiveness showed a 13.52% difference between the optimistic and pessimistic models, indicating that CEA results were sensitive to the choice of survival function.

**Table 2:**
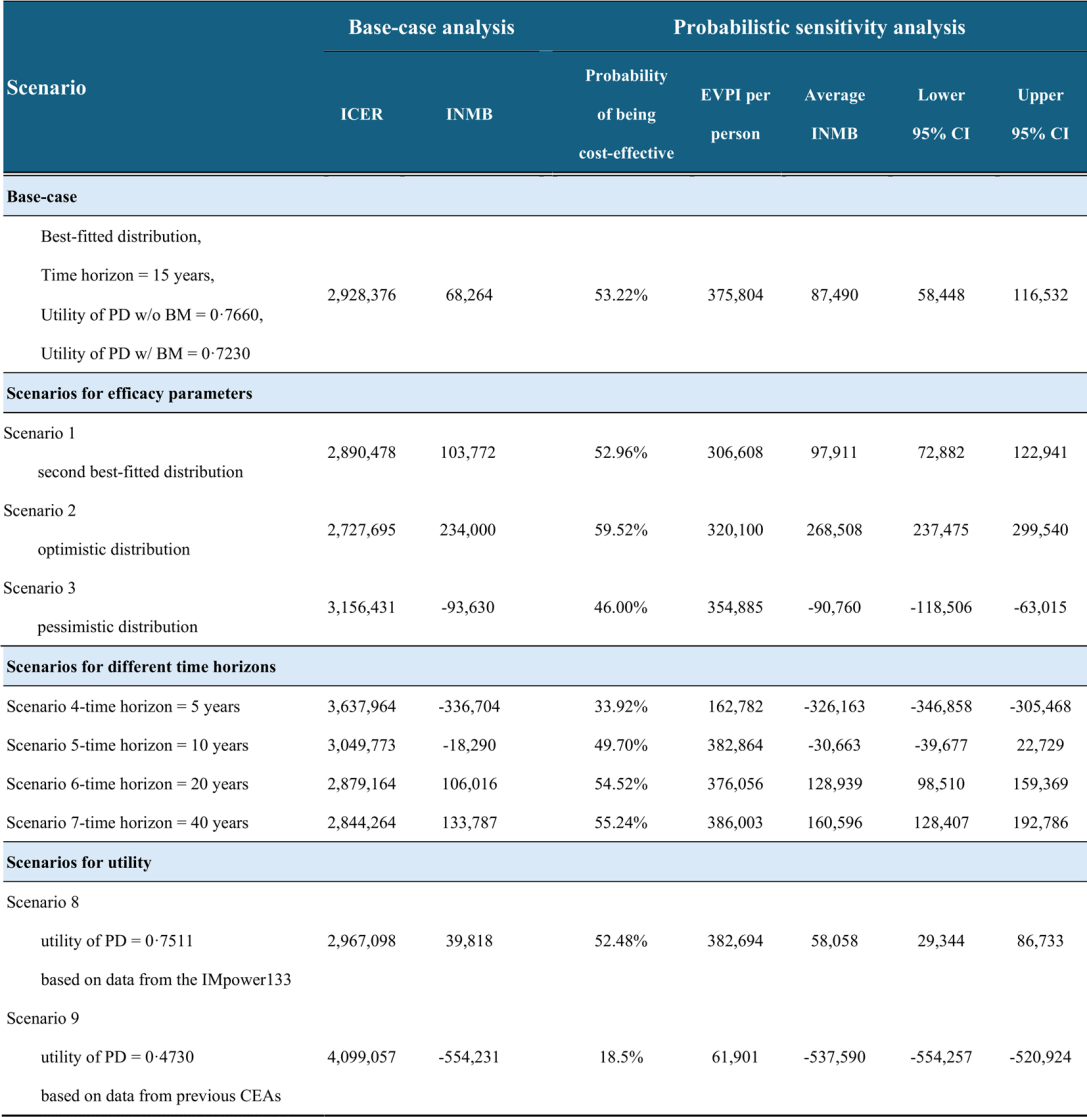
Results of scenario analysis. ICER: incremental cost-effectiveness ratio. INMB: incremental net monetary benefit. EVPI: expected value of perfect information. CI: confidence interval. PD w/o BM: progressed disease without brain metastases. PD w/ BM: progressed disease with brain metastases. PD: progressed disease.

ICER: incremental cost-effectiveness ratio. INMB: incremental net monetary benefit. EVPI: expected value of perfect information. CI: confidence interval. PD w/o BM: progressed disease without brain metastases. PD w/ BM: progressed disease with brain metastases. PD: progressed disease.

Scenarios 4–7 explore the impact of the time horizon. The probabilities of the intervention being cost-effective were 33.92% and 49.70% when adopting time horizons of 5 and 10 years, respectively. Extending the time horizon increased the cost-effectiveness of atezolizumab plus chemotherapy; however, this effect diminished after 15 years of treatment.

In Scenarios 8 and 9, the PD utility is assumed to be constant and unaffected by the BM status. Both Scenario 8 (time-invariant, regardless of BM status) and our base-case model (time-varying, accounting for BM status) calculated the utility value using all participants of IMpower133. The probabilities of cost-effectiveness were similar; however, our case model had a higher INMB. Scenario 9, which referenced the utility data used in previous CEA studies, significantly reduced the probability of cost-effectiveness by 18.5%.

## Discussion

This study demonstrated that combining atezolizumab with chemotherapy is a cost-effective approach for patients with EX-SCLC without baseline BM. Adding atezolizumab led to an ICER of NT$2,928,376 per QALY (lower than the WTP threshold of NT$3,023,055) and an INMB of NT$68,264. The cost-effectiveness probability was 53.22%, highlighting the substantial uncertainty primarily driven by treatment efficacy, time horizon, and utility.

Our CEA results differ substantially from those of previous studies examining the addition of atezolizumab to chemotherapy, which reported ICERs well above WTP thresholds and low cost-effectiveness probabilities (0–6%)^6–10^. A key advancement in our analysis was identifying EX-SCLC patients without baseline BM, who are more likely to benefit from the intervention, and incorporating BM into the multistate Markov model to capture variations in treatment efficacy and disease development. While earlier studies used shorter time horizons^6,8,9^, we applied a 15-year horizon aligned with Taiwan’s average life expectancy, extending to 20 years to account for more than 99% of deaths and 40 years for younger patients. This approach, consistent with the NICE recommendation for longer horizons to reflect the long-term benefits of immunotherapies, provides a more accurate assessment of atezolizumab^14^.

Our study showed that BM status plays a crucial role in influencing the efficacy, utility, and cost-effectiveness of CEAs. The efficacy of atezolizumab for brain tumors remains uncertain because of limited data on its ability to cross the blood-brain barrier (BBB)^15^. While some studies have suggested that the BBB may become compromised in BM^16^, the IMpower133 trial showed no significant OS benefit in patients with baseline BM (HR: 0.96, 95% CI = 0.46–2.01). In contrast, patients without baseline BM had a greater OS benefit (HR: 0.74, 95% CI = 0.58–0.94)^4^.

Beyond efficacy, BM reduces utility due to neurological impairment and increases healthcare costs due to more intensive treatments^12^. Ignoring BM in CEA may lead to differing cost-effectiveness conclusions. In Scenario 8, equating utility values by using the mean average from the IMpower133 trial significantly reduced INMB. Scenario 9 applied utility values from previous studies that did not account for BM heterogeneity, resulting in a negative INMB and contradicting the cost-effectiveness findings of our base case. These assumptions, coupled with imprecise BM cost estimates, caused a substantial increase in the ICER and decreased the likelihood of atezolizumab being cost-effective by 18.5%. These results highlight the critical need to consider the BM status in CEAs for accurate economic evaluations and call for more exact modeling of BM’s impact of BM in subsequent research.

One key strength of this study was the use of the IPD from the IMpower133 trial, which allowed us to account for heterogeneity in utility levels and time-varying effects. We focused on patients without BM at baseline and used a Markov model to determine the utility of health status for BM incidence. Access to the IPD also enabled us to capture the utility gains from the treatment response during the PF state. In contrast, previous CEAs have often assigned a constant utility value for the entire PF period^6–10^, potentially overlooking differences in drug responses. Our approach acknowledges the major difference between the discomfort caused by the tumor burden at baseline and the relief experienced following first-line treatment. In our study, we meticulously accounted for the disutility caused by AEs during the PF state when patients received first-line treatment. Using a GEE approach, we assessed the impact of specific AEs on utility by adjusting for drug response and the occurrence of other AEs^17^. This approach is particularly beneficial because it handles repeated measures in longitudinal data, offering robust estimates of utility changes associated with specific AEs. Our study captured the time-varying effects of treatment response, AE incidence, and withdrawal due to intolerance in the PF state. By analyzing the response and AE incidences per cycle, we estimated each cycle’s weighted utility and disutility over time. We also considered treatment withdrawal owing to intolerance, a factor often omitted in previous CEAs, potentially leading to inflated medication costs, especially for expensive treatments such as atezolizumab. These enhancements yield more accurate estimates of costs and utilities, thereby improving the precision of CEA.

These strengths enhance the robustness of our findings; however, certain limitations remain. The first limitation arises from the Markov model’s memory deficiency, which limits its ability to incorporate prior states^18^. To address this, we implemented time-dependent transition probabilities, allowing state duration to influence transitions; however, this approach does not completely overcome the memoryless assumption. The second limitation concerns long-term extrapolation from the IMpower133 trial data, where the small sample size for certain transitions resulted in unstable survival curves and potential bias. We addressed this issue by using the best-fitting model and conducting scenario analyses for greater robustness. The third limitation involves estimating the disutility of AEs using a small sample size, which leads to statistically insignificant AE coefficients. Future research should incorporate real-world data to enhance statistical power. Fourth, the low proportion of patients with baseline BM in the IMpower133 trial (9%, *n* = 35) limits our ability to conduct a simultaneous CEA for this subgroup. The fifth limitation relates to using claims data to determine progression, based on the initiation of second-line therapy or new metastatic diagnoses, which may overlook tumor growth as an independent progression factor, potentially extending PFS and inflating PF costs^19^. In addition, the utility values were mapped from EQ-5D-5 L to EQ-5D-3 L to ensure consistency with the established cost-effectiveness modeling standards. While this approach may not fully capture the quality-of-life impact of brain metastases, it remains the most appropriate and widely accepted method in accordance with NICE-recommended practice^20^. Furthermore, Taiwan’s NHI only covers carboplatin for patients with EX-SCLC and renal impairment, which is not aligned with our study population. To avoid overestimating the cost of carboplatin, we estimated its cost based on that of cisplatin within our study population, adjusting it according to the carboplatin-to-cisplatin cost ratio. Finally, cost-effectiveness reflects prices at a fixed time point. No future price reductions were assumed in our analysis; however, the price of novel therapies typically declines to a greater extent than that of standard treatments. The estimated cost-effectiveness is therefore likely conservative and may improve over time.

In conclusion, adding atezolizumab to chemotherapy appears to be cost-effective compared to chemotherapy alone for EX-SCLC without baseline BM under Taiwan’s NHI system, though a high level of uncertainty remains. These findings underscore the importance of accounting for structural assumptions and decision uncertainty—particularly regarding BM status and time horizon—in CEAs of high-cost therapies. Future evaluations should incorporate subgroup heterogeneity and explicitly characterize uncertainty to ensure robust and reliable conclusions.

## Methods

### Analytical model structure

We developed a four-state Markov model over 15 years. The intervention was atezolizumab combined with chemotherapy (carboplatin and etoposide), compared with chemotherapy alone, as in the IMpower133 trial. The model used a 3-week cycle and applied a 3% annual discount rate to both health outcomes and direct medical costs^21^. Utility values and transition probabilities were estimated using individual patient data (IPD) from the IMpower133 trial. This study followed the guidelines of the Professional Society for Health Economics and Outcomes Research^22^ and adhered to the Consolidated Health-Economic Evaluation Reporting Standards (CHEERS). Detailed information is provided in Supplementary Material 2.

### Target population

Our study focused on adults with EX-SCLC without BM at the initiation of first-line therapy. The target population was selected from the phase III IMpower133 trial^4^, with 201 patients randomly assigned to the intervention group (atezolizumab plus chemotherapy) and 202 to the comparator group (chemotherapy alone). For further analysis, IPD from IMpower133 were accessed using the Vivli data-sharing platform^4,23^. The key inclusion criteria were as follows: adults, Eastern Cooperative Oncology Group performance status score of 0–1, and no prior treatment for EX-SCLC^4^.

### Markov model

**Fig. 2:**
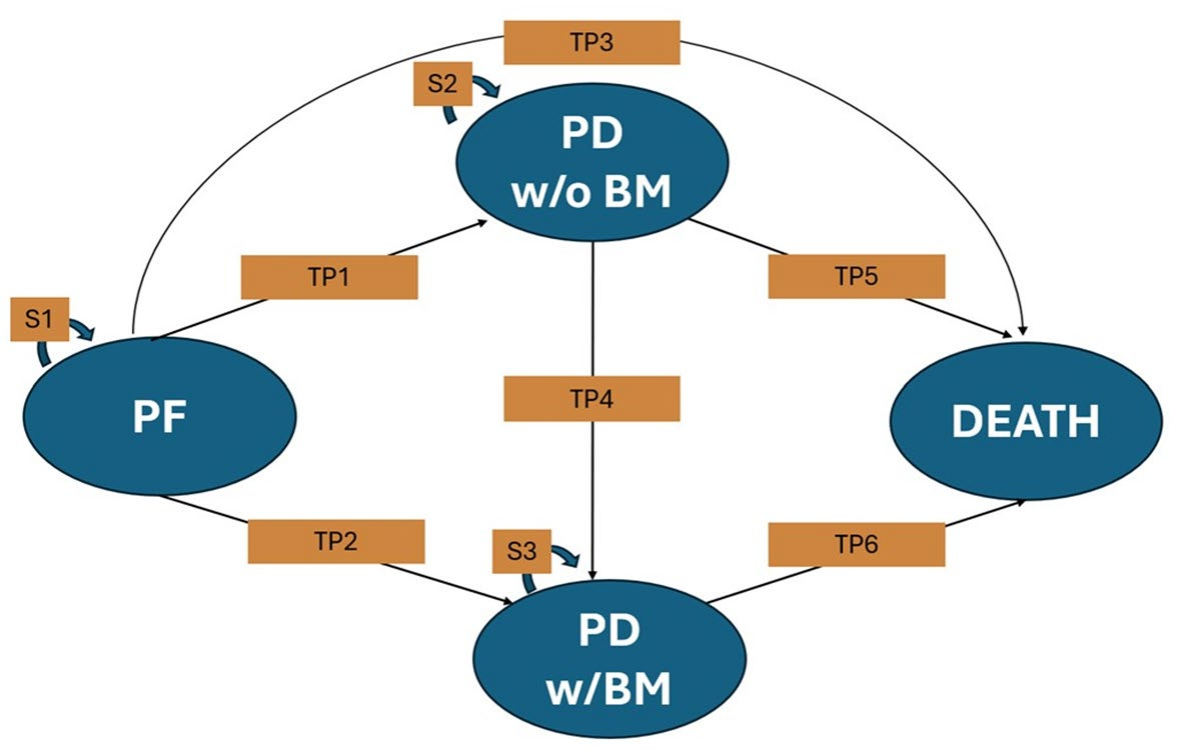
Markov state transition model. PF: progression-free. BM: brain metastases. PD w/ BM: progressed disease with brain metastases. PD w/o BM: progressed disease without brain metastases. TP1: transition probability from progression-free state to progressed disease without brain metastases.TP2: transition probability from progression-free state to progressed disease with brain metastases. TP3: transition probability from progression-free state to death. TP4: transition probability from progressed disease without brain metastases to progressed disease with brain metastases.TP5: transition probability from progressed disease without brain metastases to death. TP6: transition probability from progressed disease with brain metastases to death. S1: stay at progression-free state. S2: Stay at progressed disease without brain metastases. S3: Stay at progressed disease with brain metastases.

Our Markov state transition model consisted of four health states (Fig. 2): progression-free (PF), progressive disease (PD) without BM, PD with BM, and death. PD without BM refers to disease progression without BM, whereas PD with BM indicates disease progression accompanied by at least one BM. Once individuals enter either PD state, they cannot revert to the PF state; similarly, individuals in the PD with BM state cannot transition back to the PD without BM state. Our analytical model was implemented using TreeAge Pro Healthcare version 2024 (TreeAge Software LLC, Williamstown, MA, USA).

### Intervention and comparator

The intervention and comparator treatment strategies were based on the IMpower133 trial protocol. The intervention regimen consisted of induction and maintenance phases. During the induction phase, comprising four 21-day cycles, the patients received atezolizumab (1,200 mg/cycle) in combination with chemotherapy: etoposide (100 mg/m^2^ on days 1–3) and carboplatin (AUC 5 mg/mL/min). In the maintenance phase, patients received atezolizumab (1,200 mg/cycle every 21 days) until intolerance or disease progression. The control group received the chemotherapy regimen only during the induction phase^4^.

### Outcomes, cost-effectiveness measures, and willingness-to-pay

Outcomes included QALYs and life-years (LYs). The primary cost-effectiveness measure was the incremental cost-effectiveness ratio (ICER), representing the additional cost per QALY gained for atezolizumab plus chemotherapy compared with chemotherapy alone. We also reported the incremental net monetary benefit (INMB), which translates health gains into monetary terms by applying the willingness-to-pay (WTP) threshold and subtracting incremental costs. A positive INMB indicates that the intervention is cost-effective at the specified WTP. Unlike ICER, which reports cost per QALY, INMB summarizes results as a net monetary gain, making interpretation and cross-intervention comparison more straightforward. In line with WHO-recommended practice and previous literature, the WTP threshold was set at three times the 2023 GDP per capita (NT$3,023,055) per QALY gained^24^.

### Model input parameters

#### Transition probability

To identify the transition pathways and structure the time-to-transition data for each health state, the IPD from the IMpower133 trial were accessed. The ***mstate*** package in R (version 4.2.2; R Foundation for Statistical Computing, Vienna, Austria) was employed under the multistate model framework using the “clock reset” approach. Six standard parametric models—exponential, Weibull, Gompertz, log-logistic, log-normal, and generalized gamma distributions—as recommended by the National Institute for Health and Care Excellence (NICE)^14^ were fitted to the six transition pathways in the Markov model for each treatment arm (Fig. 2) using the ***flexsurvreg*** package. Model selection was based on the Akaike Information Criterion (AIC), Bayesian Information Criterion (BIC), and visual assessment (Supplementary Material 3). The best-fitting parametric model was used to estimate the time-varying state-transition probabilities.

### Adverse events

Our CEA model included treatment-related adverse events (AEs) of grade 3 and higher, with an incidence exceeding 2%. AEs in the IMpower133 trial were grouped into three categories based on clinical management: leukocyte deficiencies (e.g., neutropenia, reduced neutrophil count, leukopenia, decreased white blood cell count, and febrile neutropenia), anemia, and platelet deficiencies (e.g., thrombocytopenia and reduced platelet count).

### Utility/Disutility

Utility values were calculated using the 5-level version of the EuroQol five-dimension questionnaire (EQ-5D-5 L) results in the individual patient data of the IMpower133 trial. Following guidance from the NICE Decision Support Unit, the validated mapping algorithm developed by van Hout et al. was applied to convert the 5-level version responses into 3-level version utility values for use in our analysis^20^. Based on the literature and IPD from the IMpower133 trial, utility in the PF state was found to be significantly improved after a positive treatment response. Therefore, the mean utility values were differentiated according to the treatment response, assuming that the utility would change before and after the response.

The occurrence of AEs influences the utility values for each PF cycle. To account for this, generalized estimating equations (GEE) were used to conduct a utility variation analysis^25,26^, estimating PF utility while adjusting for AE-associated disutility. This allowed us to estimate PF utility independently of AE incidence in our analytical model. The calculated utility values were 0.74 for PF before the response and 0.81 for PF after the response. For patients with PD, utility values were set at 0.76 without BMs and 0.72 with BMs. The disutility values were − 0.0005 for leukocyte deficiency, − 0.0124 for anemia, and − 0.0692 for platelet deficiency.

In the analytical model, the total estimated PF utility for each cycle was weighted by the proportion of treatment responses observed in each cycle of the IMpower133 trial. To account for varying durations of AEs, IPD from the IMpower133 trial were used to track AE onset and resolution, allowing for the estimation of time-varying AE incidences for each treatment cycle.

### Medical cost

To estimate the direct medical costs of EX-SCLC, we drew on three nationwide databases (Supplementary Material 4)^27,28^: the Taiwan Cancer Registry (up to 2021), the National Health Insurance Research Database (NHIRD, up to 2022), and the National Death Registry (up to 2021). With Taiwan’s single-payer NHI program covering nearly the entire population, the NHIRD captures almost all medical utilization and expenditures, except for out-of-pocket expenses. By linking these claims data with detailed cancer diagnosis and staging information from the Taiwan Cancer Registry, as well as mortality data from the National Death Registry, we achieved a comprehensive, population-based estimation of direct medical costs.

From these datasets, patients were identified using a selection algorithm aligned with the IMpower133 trial, including adult patients with confirmed EX-SCLC (ICD-10: C33.9 or C34.X; histology: 8041–8043; TNM: M1 or T3/T4) diagnosed between January 1, 2015, and December 31, 2019. Patients with missing diagnostic data, other malignancies, or catastrophic illnesses were excluded. Patients without BM at baseline were identified using BM diagnosis and treatment data from the NHIRD. Furthermore, patients not receiving platinum-based first-line therapy were excluded, resulting in a final cohort of 1,335 patients for cost estimation across Markov model health states.

The PF state begins on the date of EX-SCLC diagnosis, as recorded in the Taiwan Cancer Registry, and ends on the earliest of the following dates: disease progression, death, or follow-up completion. Disease progression was defined as the initiation of second-line chemotherapy or the identification of new organ metastases based on diagnostic codes, radiotherapy, or surgery records. Death dates were obtained from the National Death Certification Registry. Supplementary Material 5 summarizes the cost parameters derived from NHI-covered real-world data on medication (first- and second line), non-medication, and supportive care costs during the final month of life^29^, stratified by health status.

Since the NHI reimburses the same price for a given drug across different indications and had not covered atezolizumab for EX-SCLC during our analysis period, we calculated the cost of atezolizumab using a bottom-up approach, referencing its listing price for other indications. The medication cost for atezolizumab was NT$83,258 per cycle, based on the 2024 reimbursement rates. As carboplatin is reimbursed by the NHI only for patients with renal impairment or intolerance to cisplatin, the average cost per cycle was derived from cisplatin costs, adjusted according to the carboplatin-to-cisplatin ratio. This ratio was calculated for a hypothetical 65-year-old, 70-kg individual with a body surface area of 1.6 m^2^ and a creatinine clearance of 63 mL/min in Taiwan^30^. Medication costs were adjusted according to the proportion of patients remaining on treatment during each cycle of the IMpower133 trial. AE management costs were included in the PF state to assess the impact of AEs on initial treatment expenses. The average AE management costs per event were derived from the NHIRD, and the expected AE costs were estimated based on the time-varying AE probabilities reported in the IMpower133 trial. Non-medication costs per cycle during the PF state included tumor surgery, radiological diagnostics, and radiotherapy, with other health resource utilization categorized separately.

For medication costs in both PD with and without BM, topotecan was used as the subsequent chemotherapy, as nearly half of the patients with disease progression in the IMpower133 trial received this treatment (51.7% in the intervention group and 42.3% in the comparator group)^4^. Non-medication costs for the PD states were estimated similarly to those for the PF state. Supportive care costs were calculated as the average expenses incurred by patients 30 days prior to death, excluding expenses related to active treatments, such as chemotherapy, radiotherapy, or surgical procedures.

Under the global budget system, Taiwan’s NHIA employs a point-based fee schedule for reimbursements, applying a conversion factor to discount the points claimed for non-medication services. In 2023, this conversion factor was set at 0.9198.

### Uncertainty analysis

To ensure the robustness of our findings and account for uncertainty in the base-case results, deterministic and probabilistic sensitivity analyses (DSA and PSA, respectively) were conducted. In the DSA, transition probability-related survival function parameters varied within their 95% confidence intervals. The conversion factor was set to its maximum and minimum values across regions and hospital levels in 2023, while the other parameters were varied by 25% from the baseline value. For the probabilistic sensitivity analysis (PSA), 5,000 Monte Carlo simulations were conducted to characterize parameter uncertainty. Cost-effectiveness acceptability (CEA) curves were generated, and the expected value of perfect information (EVPI) was estimated. EVPI, defined as the difference between the expected net monetary benefit (NMB) under perfect information and the maximum expected NMB under current uncertainty, captures both the probability of making an incorrect decision—driven by the position of the mean incremental net benefit (INB) relative to the decision threshold—and the monetary consequences of such an error, scaled by the cost-effectiveness threshold.

Nine scenario analyses were conducted to explore the impact of different assumptions on the CEA results. These scenarios addressed variations in efficacy (Scenarios 1–3), time horizon (Scenarios 4–7), and the utility of PD (Scenarios 8–9). In Scenario 1, the second-best-fit model according to AIC and BIC values was used for each transition. Scenarios 2 (optimistic) and 3 (pessimistic) employed, respectively, distributions with the most and least favorable long-term survival probabilities among the three models with the lowest AIC and BIC values. Scenarios 4–7 explored different time horizons. In Scenario 8, the utility of PD was estimated for all participants in the IMpower133 trial (utility = 0.7511), regardless of BM status. Scenario 9 adopted the utility of PD from previous CEA studies (utility = 0.473). For each scenario, complete CEA, including DSA and PSA, was performed.

### Model validation

The Assessment of the Validation Status of Health-Economic Decision Models^31^ was used to validate the model, addressing five aspects: conceptual model, input data, computerized model, operational validation, and other validation techniques. Our conceptual model was adapted from the frameworks developed by NICE^14^ and the Canadian Agency for Drugs and Technologies in Health^32^, incorporating the natural development of BM in patients with EX-SCLC. Conducting a partitioned survival model concurrently to explore model uncertainty was deemed infeasible because it lacked the flexibility to reflect the complexity of disease states in our Markov model. Two clinical experts reviewed the transition probability-related survival curves, quality-of-life data, AE incidence, and direct medical costs estimated from the NHIRD. To avoid logical errors, the TreeAge Pro Healthcare model and SAS and R programs were reviewed by two researchers from our team.

## Supplementary Information

Below is the link to the electronic supplementary material.

Supplementary Material 1

Supplementary Material 2

Supplementary Material 3

Supplementary Material 4

Supplementary Material 5

## Data Availability

The anonymous individual patient data regarding effectiveness, utility, and adverse event incidences are owned by Roche and can be accessed through Vivli, Inc. Data for cost estimation is available from the Ministry of Health and Welfare in Taiwan. However, there are restrictions on the availability of this data, as it was obtained under a license for the current study and is not publicly accessible.
