# Correction: Modeling brain metastases in cost effectiveness analysis of atezolizumab for extensive stage small cell lung cancer

**DOI:** 10.1038/s41598-026-55710-7

**Published:** 2026-06-16

**Authors:** Hsiao-Ling Chen, Chen-Han Chueh, Wei-Ming Huang, Shu-Hua Chan, Hsiao-Hsiang Cheng, Shao-Chin Chiang, Chiao-En Wu, Yu-Wen Wen, Yi-Wen Tsai

**Affiliations:** 1 Institute of Health and Welfare Policy, National Yang Ming Chiao Tung University, No. 155, Sec.2, Linong Street, Taipei City, Taiwan; 2 Herbert Wertheim School of Public Health and Human Longevity Science, University of California San Diego, La Jolla, San Diego, CA USA; 3 Medical AI Development Center, Taipei Veterans General Hospital, Taipei, Taiwan; 4 Department of Pharmacy, National Yang Ming Chiao Tung University, Taipei, Taiwan; 5 Department of Oncology and Hematology, Koo Foundation Sun Yat-Sen Cancer Center, Taipei, Taiwan; 6 Department of Pharmacy, Koo Foundation Sun Yat-Sen Cancer Center, Taipei, Taiwan; 7 Division of Hematology-Oncology, Department of Internal Medicine, Chang Gung Memorial Hospital at Linkou, Chang Gung University College of Medicine, Taoyuan, Taiwan; 8 Division of Hematology-Oncology, Department of Internal Medicine, New Taipei Municipal TuCheng Hospital, New Taipei, Taiwan; 9 Department of Biomedical Sciences, Chang Gung University, No.259, Wenhua 1St Rd., Guishan Dist, Taoyuan City, Taiwan

Correction to: *Scientific Reports* 10.1038/s41598-025-22966-4, published online 10 November 2025

The original version of this Article contained errors in Tables 1 and 2, where truncated screenshots which also included Word formatting symbols were inadvertently published.

The original Tables 1 and 2 and their accompanying legends appear below.

**Table 1:**
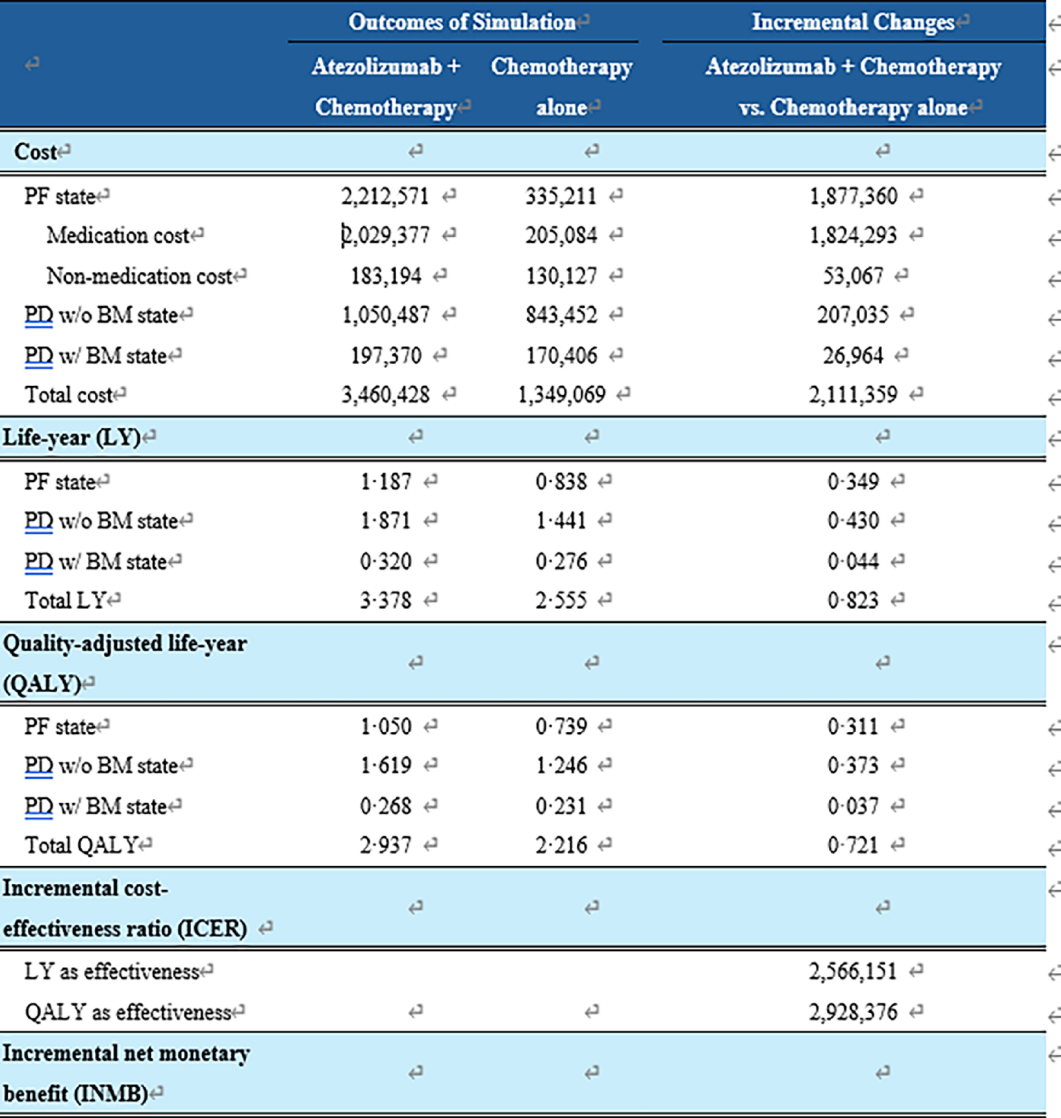
Results of the base-case analysis. PF: progression-free. PD w/o BM: progressed disease without brain metastases. PD w/ BM: progressed disease with brain metastases. Costs listed in New Taiwan Dollar (NTD). Medication cost: include cost from atezolizumab and chemotherapy. non-Medication cost: include cost form surgery, therapy for adverse events, radiotherapy and other health resource cost for SCLC. PD state cost includes subsequent therapy, surgery, radiotherapy and other health resource cost for SCLC

**Table 2:**
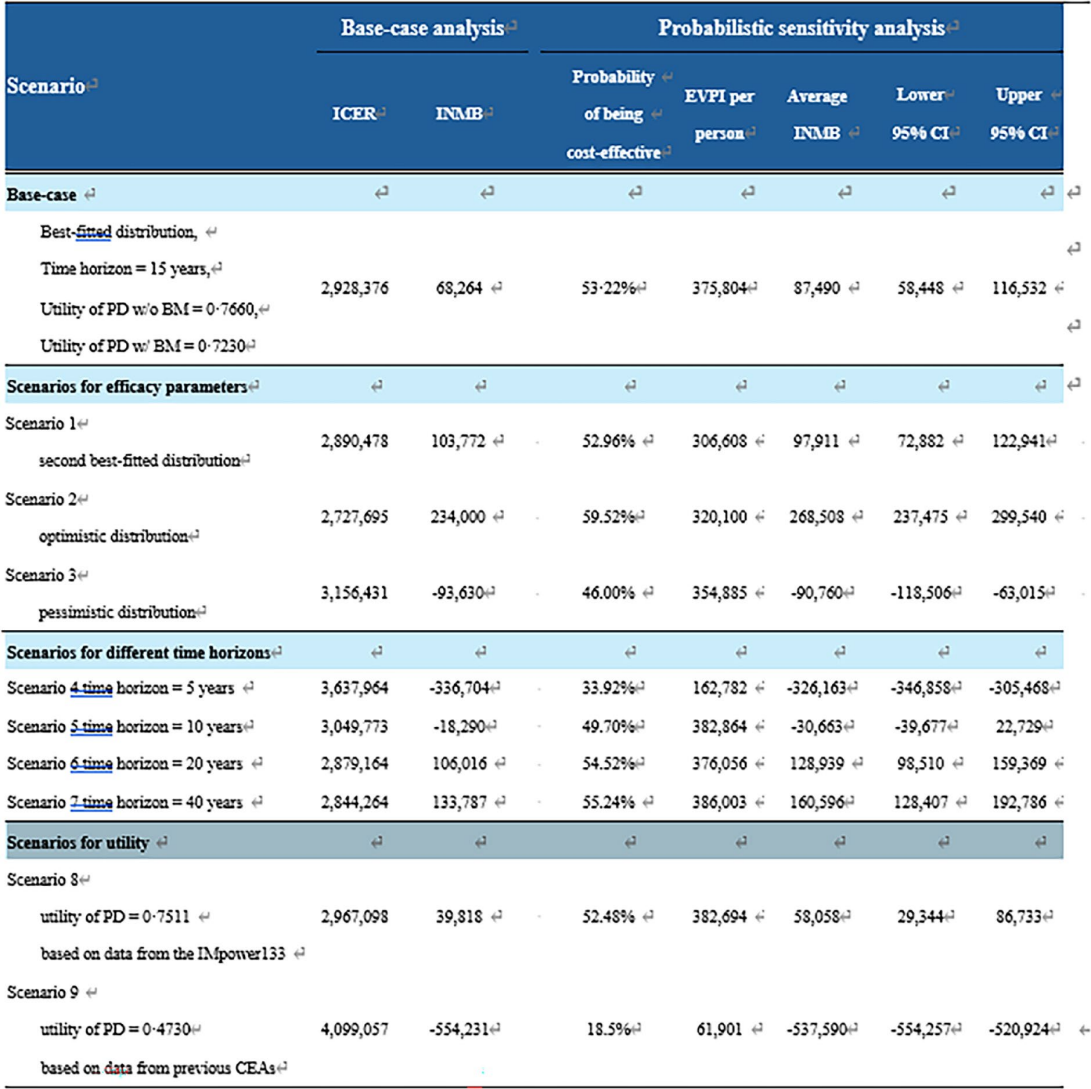
Results of scenario analysis. ICER: incremental cost-effectiveness ratio. INMB: incremental net monetary benefit. EVPI: expected value of perfect information. CI: confidence interval. PD w/o BM: progressed disease without brain metastases. PD w/ BM: progressed disease with brain metastases. PD: progressed disease.

The original Article has been corrected.

