# Supplementary material for: Modeling brain metastases in cost effectiveness analysis of atezolizumab for extensive stage small cell lung cancer
Source: Sci Rep. 2025 Nov 10;15:39298. doi: 10.1038/s41598-025-22966-4 (PMC12603174; doi:10.1038/s41598-025-22966-4)
Supplement: Supplementary file 1 — Supplementary Material 1 [file 41598_2025_22966_MOESM1_ESM.pdf]

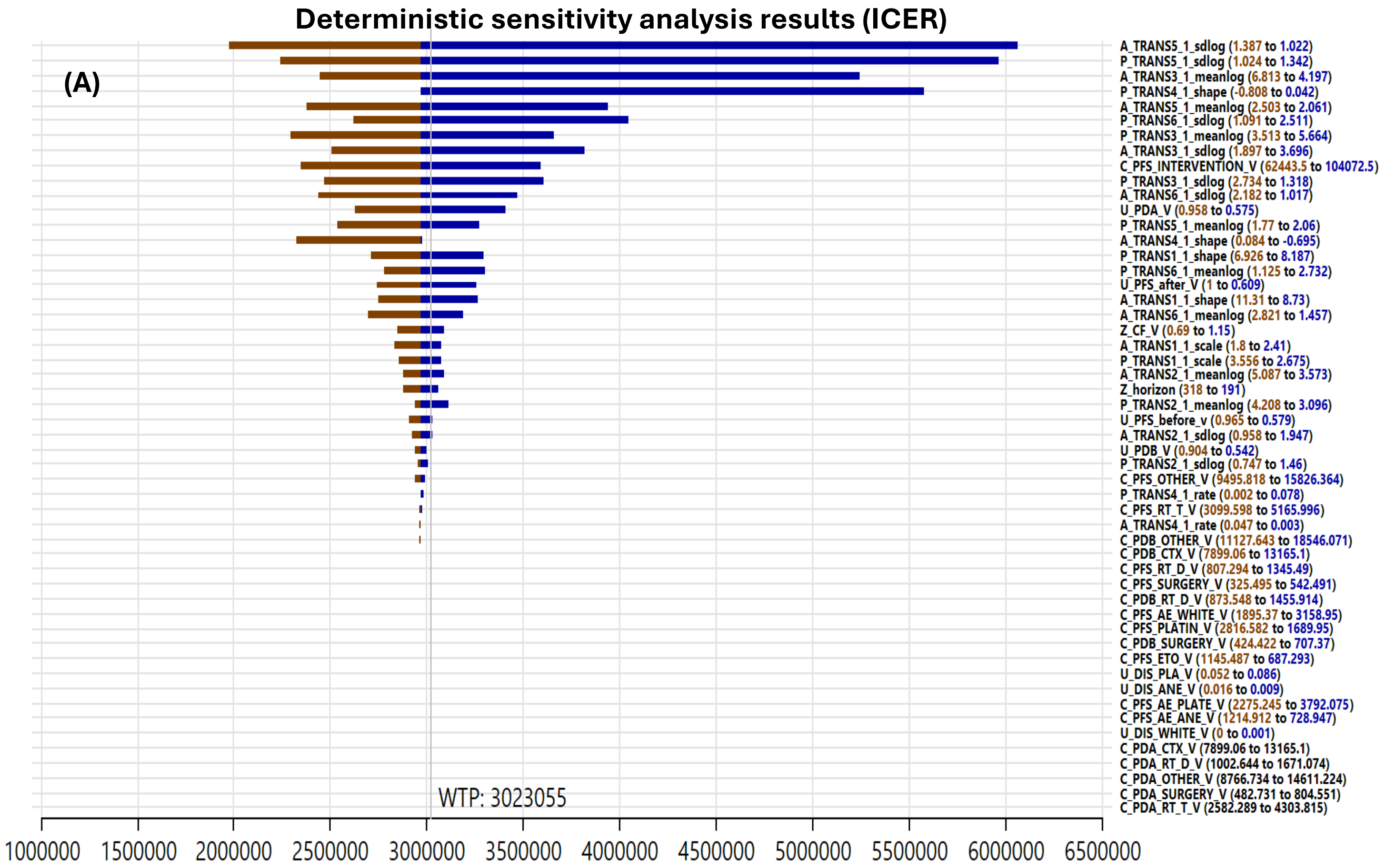

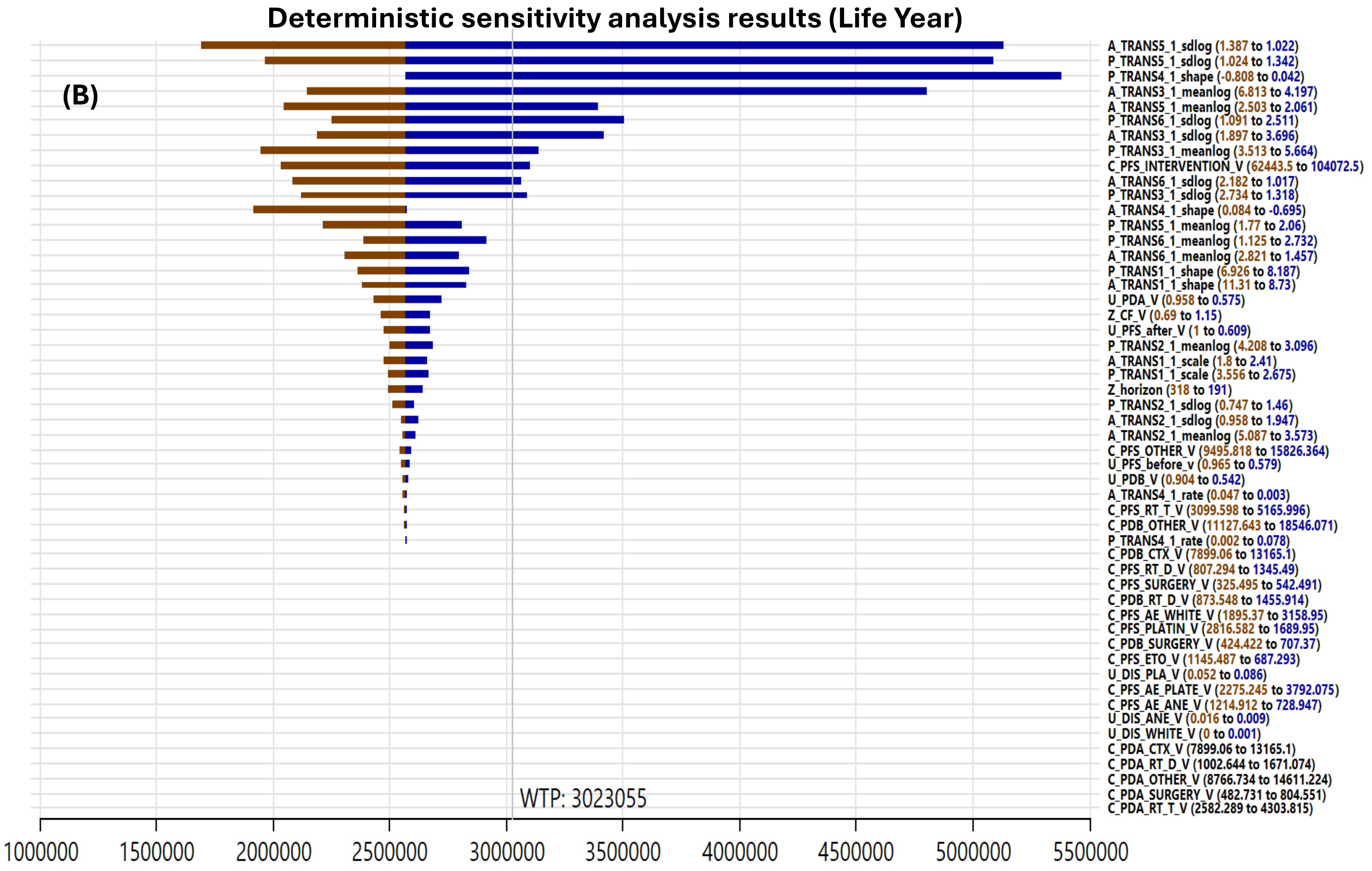

Supplement 1. Deterministic sensitivity analysis results. (A) incremental cost per additional quality-adjusted life-year (QALY) gained and (B) incremental net monetary benefit (NMB) gained for atezolizumab plus chemotherapy versus chemotherapy

The dashed line indicates the willingness to-pay (WTP) threshold of NT$3,025,055 per QALY gained. Costs listed in 2023 New Taiwan Dollar (NTD).

A: atezolizumab+ chemotherapy (platinum drugs & etoposide).

P: chemotherapy (platinum drugs & etoposide).

TRANS1_1: transition probability from progression-free state to progressed disease without brain metastasis.

TRANS2_1: transition probability from progression-free state to progressed disease with brain metastasis.

TRANS3_1: transition probability from progression-free state to death.

TRANS4_1: transition probability from progressed disease (PD) without brain metastasis to PD with brain metastasis.

TRANS5_1: transition probability from progressed disease without brain metastasis to death.

TRANS6_1: transition probability from progressed disease with brain metastasis to death.

PFS: progress free survival.

PDA: progressed disease without brain metastases.

PDB: progressed disease with brain metastases.

sdlog: standard deviation of the logarithm; meanlog: mean of the logarithm

C_PFS_INTERVENTION_V: cost of atezolizumab at PFS

C_PFS_PLATIN_V: cost of platinum drugs at PFS

C_PFS_ETO_V: cost of etoposide at PFS

C_PFS_RT_V: cost of radiotherapy at PFS

C_PFS_SURGERY_V: cost of surgery at PFS

C_PFS_AE_ANE_V : cost of anemia therapy at PFS

C_PFS_AE_WHITE_V : cost of leukocyte deficiencies at PFS

C_PFS_AE_PLATE_V : cost of platelet deficiencies at PFS

C_PFS_OTHER_V: cost of other health resource for SCLC at PFS

C_PDA _CTX_V : cost of subsequent therapy at PDA

C_PDB_CTX_V : cost of subsequent therapy at PDB

C_PDA_RT_V : cost of radiotherapy for SCLC at PDA

C_PDB_RT_V : cost of radiotherapy for SCLC at PDB

C_PDA_SURGERY_V : cost of surgery for SCLC at PDA

C_PDB_SURGERY_V : cost of surgery for SCLC at PDB

C_ PDA_OTHER_V: cost of other health resource for SCLC at PDA

C_ PDB_OTHER_V: cost of other health resource for SCLC at PDB

U_PFS_before_V: utility at PFS before treatment response

U_PFS_after_ V: utility at PFS after treatment response

U_DIS_ANE_V: disutility due to anemia

U_DIS_WHITE_V: disutility due to leukocyte deficiencies

U_DIS_PLATE_V: disutility due to platelet deficiencies

U_PDA_V: utility of PDA

U_PDB_V: utility of PDB

Z_CF_V : conversion factor of National Health Insurance

Z_horizon: time horizon
