# Supplementary material for: Modeling brain metastases in cost effectiveness analysis of atezolizumab for extensive stage small cell lung cancer
Source: Sci Rep. 2025 Nov 10;15:39298. doi: 10.1038/s41598-025-22966-4 (PMC12603174; doi:10.1038/s41598-025-22966-4)
Supplement: Supplementary file 3 — Supplementary Material 3 [file 41598_2025_22966_MOESM3_ESM.pdf]

Supplement3. Akaike Information Criterion (AIC) & Bayesian Information Criterion (BIC) for Each State Transition

atezolizumab+chemotherapy			chemotherapy			base case (best fit)	second best fit	optimistic	pessimistic
	AIC	BIC		AIC	BIC				
**TP1: transition probability from progress free state to progress disease without brain metastasis**									
Weibull	971.32	977.75	Weibull	938.07	944.48				
Exponential	975.6199	978.8348	Exponential	963.4347	966.6442				
Log-normal	924.1109	930.5408	Log-normal	871.1938	877.6128		v		v
Log-logistic	922.3012	928.7311	Log-logistic	851.4951	857.9141	v		v	
Gompertz	975.6126	982.0425	Gompertz	965.2449	971.6639				
Generalized gamma	931.78	941.43	Generalized gamma	879.77	889.40				
**TP2: transition probability from progress free state to progress disease with brain metastasis**									
Weibull	191.20	197.63	Weibull	178.06	184.48				
Exponential	190.2446	193.4595	Exponential	180.7364	183.9459			v	
Log-normal	185.9937	192.4235	Log-normal	172.1763	178.5953	v			
Log-logistic	189.9562	196.3861	Log-logistic	175.9393	182.3583		v		v
Gompertz	191.7487	198.1786	Gompertz	182.5136	188.9326				
Generalized gamma	189.134	198.7788	Generalized gamma	175.3018	184.9303				
**TP3: transition probability from progress free state to death**									
Weibull	246.16	252.59	Weibull	200.84	207.26				
Exponential	246.8244	250.0393	Exponential	198.8383	202.0478				v
Log-normal	246.1235	252.5534	Log-normal	199.1734	205.5924	v		v	
Log-logistic	245.9778	252.4077	Log-logistic	200.3326	206.7516		v		
Gompertz	246.0827	252.5126	Gompertz	200.2244	206.6434				
Generalized gamma	247.92	257.56	Generalized gamma	201.57	211.20				
**TP4: transition probability from progress disease without brain metastasis to progress disease with brain metastasis**									
Weibull	57.3632	63.06726	Weibull	32.21602	38.11354				
Exponential	57.22887	60.0809	Exponential	32.08024	35.029				v
Log-normal	56.6935	62.39756	Log-normal	31.939	37.83652		v		
Log-logistic	57.3128	63.01686	Log-logistic	32.20556	38.10308				
Gompertz	53.68565	59.38971	Gompertz	29.59398	35.4915	v		v	
Generalized gamma	58.93	67.49	Generalized gamma	34.07	42.91				
**TP5: transition probability from progress disease without brain metastasis to death**									
Weibull	691.67	697.37	Weibull	796.59	802.49				
Exponential	689.7859	692.6379	Exponential	794.758	797.7067				
Log-normal	673.0297	678.7337	Log-normal	776.9606	782.8582	v			v
Log-logistic	674.2018	679.9058	Log-logistic	775.8767	781.7743		v	v	
Gompertz	682.8743	688.5783	Gompertz	786.7789	792.6765				
Generalized gamma	677.32	685.88	Generalized gamma	780.92	789.77				
**TP6: transition probability from progress disease with brain metastasis to death**									
Weibull	115.3179	117.3094	Weibull	95.72645	97.50719				
Exponential	114.6189	115.6147	Exponential	98.60539	99.49576				
Log-normal	112.5409	114.5324	Log-normal	91.17476	92.9555	v			v
Log-logistic	113.1957	115.1871	Log-logistic	91.65867	93.43941		v	v	
Gompertz	112.7781	114.7696	Gompertz	87.96733	89.74807				
Generalized gamma	115.07	118.06	Generalized gamma	93.97	96.64				

optimistic: the most optimistic long-term survival scenario among the top three models with the lowest AIC and BIC scores.
pessimistic: the most pessimistic long-term survival scenario among the top three models with the lowest AIC and BIC scores.
