# Supplementary material for: Modeling brain metastases in cost effectiveness analysis of atezolizumab for extensive stage small cell lung cancer
Source: Sci Rep. 2025 Nov 10;15:39298. doi: 10.1038/s41598-025-22966-4 (PMC12603174; doi:10.1038/s41598-025-22966-4)
Supplement: Supplementary file 4 — Supplementary Material 4 [file 41598_2025_22966_MOESM4_ESM.pdf]

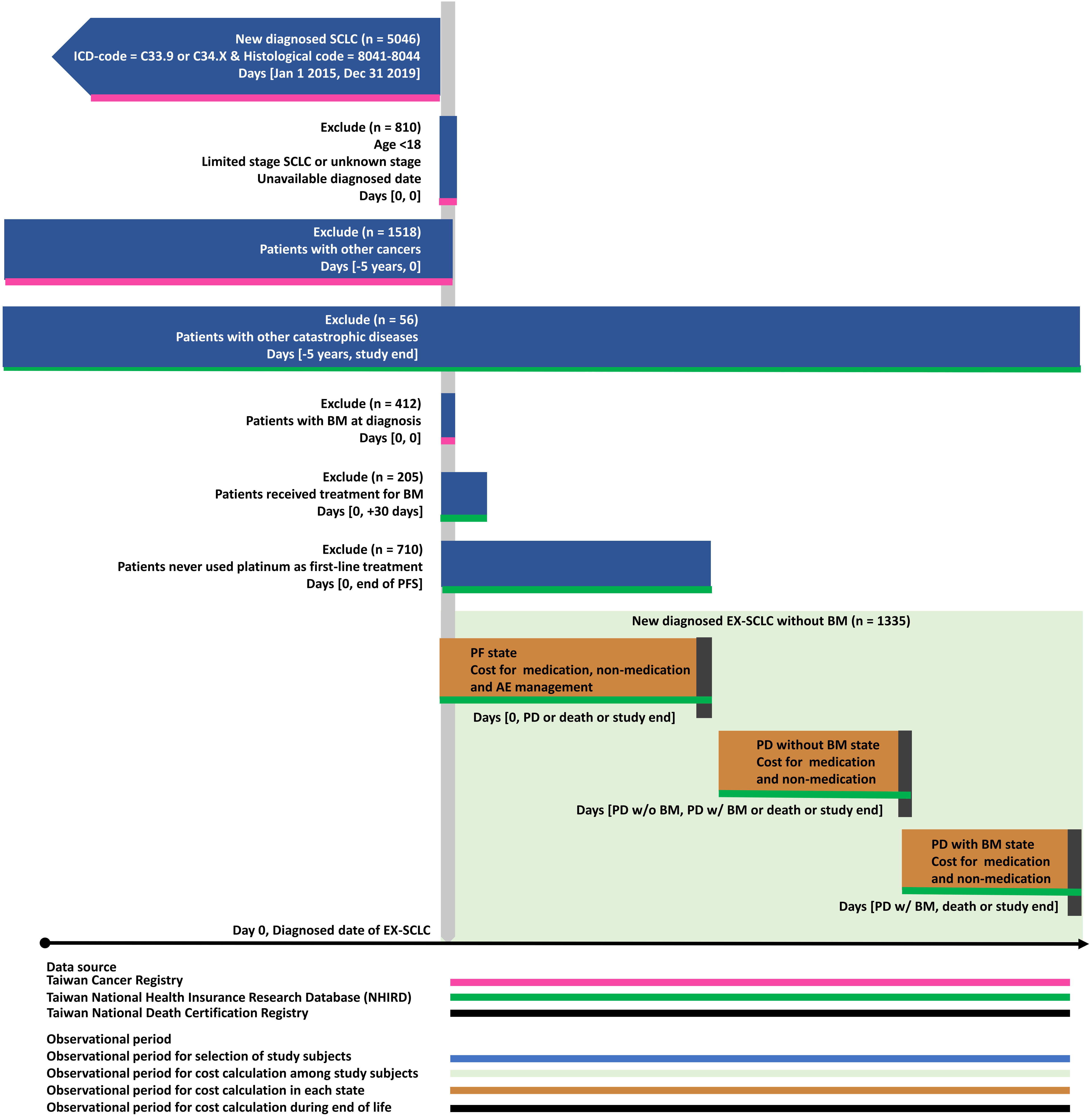

Supplement 4. Flowchart for sample selection in cost estimation. SCLC: small cell lung cancer. ICD: International Classification of Diseases. BM: brain metastases. EX-SCLC: extensive stage small cell lung cancer. PF: progression-free. PD: progressed disease. PD w/o BM: progressed disease without brain metastases. PD w/ BM: progressed disease with brain metastases.
