# Supplementary material for: Modeling brain metastases in cost effectiveness analysis of atezolizumab for extensive stage small cell lung cancer
Source: Sci Rep. 2025 Nov 10;15:39298. doi: 10.1038/s41598-025-22966-4 (PMC12603174; doi:10.1038/s41598-025-22966-4)
Supplement: Supplementary file 5 — Supplementary Material 5 [file 41598_2025_22966_MOESM5_ESM.pdf]

Supplement5. Cost parameters, baseline values, ranges, and distributions for sensitivity analyses (data from NHIRD)

Model input parameters	Estimated value	DSA		PSA		
		Range (95% CI or ± 25%)		Distribution/parameters		
**PF**						
**Medication cost**						
Atezolizumab	83258·0	62443·5	104072·5	Uniform (α, β)	62443·5	104072·5
Carboplatin	2253·3	1689·9	2816·6	Gamma (μ, SE)	2253·3	54·5
Etoposide	916·4	687·3	1145·5		916·4	59·2
**AE management**						
Leukocyte deficiency	2527·2	1895·4	3159·0		2527·2	169·0
Anemia	971·9	728·9	1214·9	Gamma (μ, SE)	971·9	90·0
Platelet deficiency	3033·7	2275·2	3792·1		3033·7	742·2
**Non-medication cost**						
Tumor surgeries	434·0	325·5	542·5		434·0	377·2
Radiological diagnosis	1076·4	807·3	1345·5		1076·4	29·5
Radiological therapy	4132·8	3099·6	5166·0	Gamma (μ, SE)	4132·8	35·2
Other health resource utilization	12661·1	9495·8	15826·4		12661·1	107·9
**PD w/o BM**						
**Medication cost**						
Subsequent chemotherapy	9398·5	7048·9	11748·1	Gamma (μ, SE)	9398·5	557·7
**Non-medication cost**						
Tumor surgeries	643·6	482·7	804·6		643·6	307·7
Radiological diagnosis	1336·9	1002·6	1671·1		1336·9	51·6
Radiological therapy	3443·1	2582·3	4303·8	Gamma (μ, SE)	3443·1	132·9
Other health resource utilization	11689·0	8766·7	14611·2		11689·0	136·1
**PD with BM**						
**Medication cost**						
Subsequent chemotherapy	8851·5	6638·6	11064·4	Gamma (μ, SE)	8851·5	676·9
**Non-medication cost**						
Tumor surgeries	565·9	424·4	707·4		565·9	201·8
Radiological diagnosis	1164·7	873·5	1455·9		1164·7	54·4
Radiological therapy	3891·4	2918·5	4864·2	Gamma (μ, SE)	3891·4	181·6
Other health resource utilization	14836·9	11127·6	18546·1		14836·9	166·2
**End of life**						
Supportive care cost	158826·4	119119·8	198533·1	Gamma (μ, SE)	158826·4	7221·5

NHIRD: National Health Insurance Research Database. DSA: deterministic sensitivity analysis. CI: confidence interval. PSA: probabilistic sensitivity analysis. PF: progression-free. AE: adverse event. PD w/o BM: progressed disease without brain metastases

* The estimated value for Atezolizumab is based on Taiwan's NHI reimbursement rates in 2024, using a bottom-up approach. Costs for other treatments were estimated using a top-down method derived from NHIRD data.
